# Distant metastasis in patients with myxofibrosarcoma

**DOI:** 10.1080/03009734.2017.1356404

**Published:** 2017-08-17

**Authors:** Hiroyuki Tsuchie, Mitsunori Kaya, Hiroyuki Nagasawa, Makoto Emori, Yasutaka Murahashi, Emi Mizushima, Naohisa Miyakoshi, Toshihiko Yamashita, Yoichi Shimada

**Affiliations:** a Department of Orthopedic Surgery, Akita University Graduate School of Medicine, 1-1-1 Hondo, Akita 010-8543, Japan;; b Department of Orthopedic Surgery, Sapporo Medical University School of Medicine, S-1 W-16, Cyuo-ku, Sapporo, Hokkaido 060-8543, Japan

**Keywords:** Lymph node, metastasis, myxofibrosarcoma

## Abstract

**Purpose:**

A clinical feature of myxofibrosarcoma is local recurrence, but knowledge about distant metastasis is sparse. We evaluated the tendency of clinical and histological features of metastasis in myxofibrosarcoma patients.

**Methods:**

Fifty-eight patients with myxofibrosarcoma were treated in our hospitals, and a total of 16 consecutive patients with distant metastases were included in this retrospective study (9 males and 7 females, with a mean age of 77 years). Because there was no patient complicated by both lung and lymph node metastases, we compared the age, sex, tumor size and location, French Federation of Cancer Centers Sarcoma Group (FNCLCC) grade, American Joint Committee on Cancer (AJCC) stage, and times of the first metastasis from the initial examination between the lung and lymph node groups. In addition, we examined factors affecting the prognosis.

**Results:**

The median follow-up period was 42.9 months (range 8–142). Eleven of 16 patients developed pulmonary metastases. The sites of extra pulmonary metastases were the lymph nodes in 5 patients, bone in 1, subcutaneous in 1, intramuscular in 1, and peritoneum in 1. The median time for patients to develop distant metastases was 17.4 months (range 0–59). The time until the onset of the first metastasis in the lung metastasis group was significantly shorter than in the lymph node group (*p* < 0.05). Also, the survival rate in the lymph node metastasis group was better than in the lung metastasis group (*p* < 0.05).

**Conclusions:**

Not only lung metastasis but also lymph node metastasis occurs frequently in myxofibrosarcoma patients. Myxofibrosarcoma with lung metastasis is more aggressive than the type with lymph node metastasis.

## Introduction

Myxofibrosarcoma is one of the most common soft-tissue sarcomas found in the extremities of older adults and accounts for approximately 20% of all soft-tissue sarcomas (1). A clinical feature of myxofibrosarcoma is local recurrence. Complete surgical resection is the standard treatment, but negative margins are difficult to obtain because myxofibrosarcoma has an unusual infiltrative growth pattern along fascial planes (2,3). Therefore, the outcomes are characterized by a high local recurrence rate, from 15% to 57%, leading to poor overall survival (4–11). Some factors of local recurrence, such as resection with positive or close margins, bone and joint involvement, and unplanned resection, have been examined in other studies (4–6,10,11). However, there has been no systematic report on metastasis despite the many reports on local recurrence.

The aim of this study was to analyze the tendency of clinical and histological variables of metastasis in myxofibrosarcoma patients. In addition, we compared the clinical features and prognosis between patients with pulmonary metastasis and lymph node metastasis.

## Patients and methods

### Subjects

Fifty-eight patients with myxofibrosarcoma were treated in our two hospitals between 1992 and 2014 (28 males and 30 females, with a mean age of 70 years). In these patients, a total of 16 consecutive patients (27.6%) had distant metastases, and they were included in this retrospective study. Information on the patients including age, sex, and anatomical location and size of the tumor, is presented in Table 1. The stage of the primary tumor was determined according to the staging system of the American Joint Committee on Cancer (AJCC), 6th edition (12). The specimens were assigned to the French Federation of Cancer Centers Sarcoma Group (FNCLCC) classification. This classification is based on the mitotic index, necrosis extension, and histological differentiation of the tumor (13). In the absence of any events, the date of the last follow-up was considered as an end-point. In our series, there was no patient complicated by both lung and lymph node metastases, and we compared the age, sex, tumor size and location, FNCLCC grade, AJCC stage, and time until the first metastasis after the initial examination between the lung and lymph node groups. In addition, we examined factors affecting the prognosis. The study was approved by the Institutional Review Board for Clinical Research at our university, and informed consent was obtained from all patients enrolled in the study.

**Table 1.** Clinical information of 16 patients with myxofibrosarcoma.

Case	Age	Sex	Tumor location	Tumor size (cm)	FNCLCC grade	AJCC stage	Margin	Treatment excluding surgery	Recurrence	Metastasis (months)^a^	Treatment for metastasis	Follow-up period (months)	Outcome
1	82	F	Thigh	10	2	2	Wide	–	+	Lung (11)	Radiation	14	DOD
2	87	F	Buttock	7	3	3	Wide	–	–	Lymph node (32)	Surgery	56	NED
3	85	M	Forearm	5	1	1	Wide	–	–	Lung (8)	–	22	DOD
4	78	F	Thigh	1.5	1	1	Non-surgery	Radiation		Lung (14)	–	16	DOD
5	61	M	Inguinal	10	1	1	Non-surgery	Radiation		Lymph node, muscle (39)	Radiation	62	AWD
6	64	M	Upper arm	10	2	2	Wide	–	+	Lymph node (13)	Radiation, chemotherapy	142	NED
7	46	M	Upper arm	7.7	1	1	Wide	–	–	Lung (12)	Surgery, chemotherapy	66	NED
8	81	M	Chest wall	7	2	2	Intraregional	Radiation	+	Lung (8)	Surgery, radiation	90	DOD
9	79	M	Lower leg	3.5	2	2	Wide	–	–	Lung, peritoneum (19)	–	20	AWD
10	69	M	Lower leg	5	3	3	Wide	Radiation	–	Lymph node (59)	Radiation, chemotherapy	77	NED
11	87	F	Forearm	10	2	2	Intraregional	–	+	Lung (12)	–	17	DOD
12	78	F	Buttock	22	1	1	Intraregional	Radiation	+	Subcutaneous, lung, bone (15)	–	23	DOD
13	87	M	Lower leg	8.9	3	4	Wide	–	–	Lung (0)	–	8	DOD
14	89	F	Lower leg	5	3	3	Wide	Radiation	–	Lymph node (21)	Surgery	31	NED
15	65	F	Inguinal	5.5	1	1	Wide	Radiation	–	Lung (9)	Chemotherapy	23	NED
16	87	M	Shoulder	12.7	1	1	Non-surgery	Radiation		Lung (6)	–	19	AWD

a Time until the first metastasis after the initial examination.

AWD: alive with disease; DOD: dead of disease; NED: no evidence of disease.

### Statistical analysis

All values are expressed as the mean ± standard deviation (SD). Student’s *t* test, the Welch *t* test, and chi-square test were used to compare the items between the two groups. The curve for overall survival was drawn according to the Kaplan–Meier method, and differences were analyzed by applying the generalized Wilcoxon test. Probability (*p*) values less than 0.05 were considered significant.

## Results

**Figure 1.**
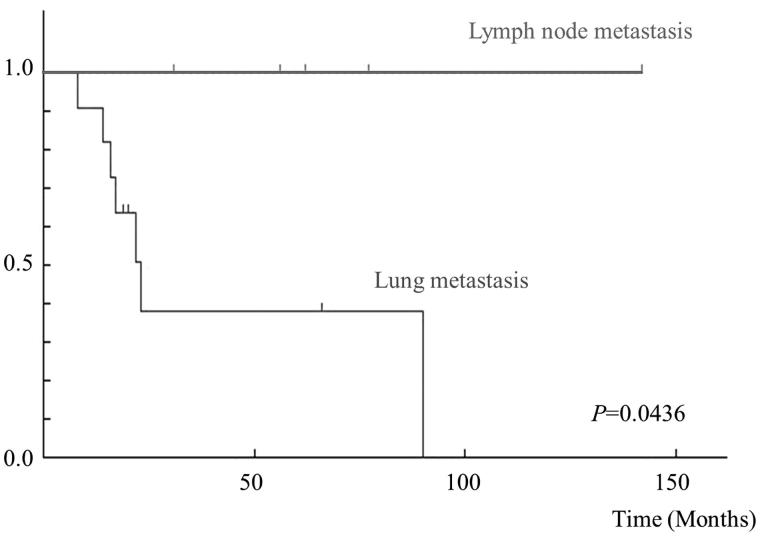
Kaplan–Meier overall survival curves according to regions of metastasis.

In the 16 patients with distant metastases, there were 9 males and 7 females, with a mean age of 77 years (range 46–89). The median follow-up period was 42.9 months (range 8–142). Five tumors were located in the upper extremities, 6 in the lower extremities, and 5 in the trunk. The mean radiological size of the tumors was 8.2 cm (range 1.5–22). A high FNCLCC grade (2 or 3) was observed in 9 (56%) patients, and a high AJCC stage (3 or 4) was observed in 4 (25%) patients. Surgery for the primary tumor was conducted in 13 patients, and an adequate tumor-free margin was obtained in 10 (76.9%). Three patients refused surgical treatment. Radiotherapy for the primary tumor was used for 8 (50%) patients. Eleven of the 16 metastasis patients (68.8% of the metastasis patients, and 17.2% of all 58 myxofibrosarcoma patients) developed pulmonary metastases. The sites of extra-pulmonary metastases were the lymph nodes in 5 of the 16 patients (31.3% of the metastasis patients, and 8.6% of all patients), bone in 1 patient, subcutaneous in 1 patient, intramuscular in 1 patient, and peritoneum in 1 patient. The median time for patients to develop distant metastases was 17.4 months (range 0–59). Treatments for metastases were surgery for 4 patients, chemotherapy for 4 patients, and radiotherapy for 5 patients. The outcome for the 16 patients was that there was no evidence of disease (NED) in 6 patients, 3 patients were alive with disease (AWD), and 7 patients died of their disease (DOD) (Table 1). In univariate analysis, the time until the first metastasis after the initial examination in the lung metastasis group (10.3 ± 5.0 months) was significantly shorter than in the lymph node group (32.8 ± 17.7 months) (*p* < 0.05) (Table 2). Moreover, the 5-year survival rate of the lung metastasis group was 25%, while that of the lymph node group was 100% (*p* < 0.05) (Figure 1).

**Table 2.** Univariate logistic regression analysis between the two groups.

	Lung group	Lymph node group	95% CI	*p* value
Age	77.7 ± 12.3	74 ± 13.1	−17.54 to 24.99	0.59
Sex			−0.844 to 0.953	0.734
Male	6	2		
Female	5	3		
Tumor location			−0.961 to 0.707	0.942
Trunk	2	3		
Extremity	3	8		
Tumor size (cm)	85.3 ± 55.0	74.0 ± 25.1	−70.85 to 93.40	0.6724
FNCLCC grade			−2.132 to 0.422	0.0538
Grade 1	6	1		
Grades 2 and 3	5	4		
AJCC stage			−2.318 to 0.791	0.1445
Stages 1 and 2	10	2		
Stages 3 and 4	1	3		
Time until first metastasis (months)	10.3 ± 5.0	32.8 ± 17.7	−40.07 to −4.804	0.0498

Values are expressed as the number of patients or mean ± SD.

95% CI = 95% confidence interval.

## Discussion

Various outcomes regarding distant metastasis in myxofibrosarcoma patients have been reported with rates of metastasis from 9.5% to 23.6% (4–11). In our study, the incidence of metastasis was quite high (27.6%). The lung has generally been thought to be the organ most frequently involved in the metastasis of soft tissue sarcoma, and certainly the lung was the most frequently involved organ in our study as well. However, lymph node metastasis was also relatively frequent in this study (8.6% of all patients). Although lymph node metastasis generally occurs in cancer, it is rare in sarcoma, affecting from 3% to 4% of all patients (14–16). A much higher propensity for lymphatic spread has been described for certain histological subtypes, including clear cell sarcoma (25% to 50%), epithelioid sarcoma (20% to 29%), synovial sarcoma (10% to 19%), and rhabdomyosarcoma (8% to 15%) (17–19). There have been few reports mentioning lymph node metastasis of myxofibrosarcoma. A review of several reports revealed an incidence of only 3% (4,8,11). As the numbers of lymph node metastasis cases in each report are very small, detailed analyses of lymph node metastasis are also very rare. In the current series of a study involving our two institutions, we found that the incidence of lymph node metastasis was higher than that in previous reports.

Because there was no patient suffering from both lung and lymph node metastases in our series, we compared evaluation items between the two groups. The time until the first metastasis after the initial examination was significantly different between the two groups, and the onset of lymph node metastasis was later than that of lung metastasis. There have been few reports on metastasis onset. Sanfilippo et al. showed that the average time until metastasis onset including all organs was 11 months (4), and Haglund et al. stated that the average time until metastasis onset not including lymph nodes was 21 months (8). In the present study, the average time until the first metastasis onset was 32.8 months and was delayed compared with previous reports. However, this difference in the timing of onset between lung and lymph node metastases was not obvious. It is therefore necessary to conduct further studies to clarify this.

Distant metastasis-free survival of myxofibrosarcoma patients was reported to be associated with mitotic activity and the margin status (20). Another study demonstrated an association between the presence of necrosis within the whole tumor and the development of distant metastasis (21). Sanfilippo et al. suggested that the histological grade of the tumor also predicted the risk of metastasis (4). In the current study, we failed to show a significant difference of such factors between lung and lymph node metastases. However, the survival rate of the lymph node metastasis group was better than that of the lung metastasis group, although only two of five lymph node metastases were surgically treated.

A limitation of this study was the small number of myxofibrosarcoma patients with distant metastasis. We could not analyze our data by multivariate analysis because of the small numbers, although various factors might lead to a bias. Myxofibrosarcoma is a relatively rare soft tissue sarcoma, and distant metastasis is even rarer. We need to perform further detailed studies with a larger number of patients with distant metastasis of myxofibrosarcoma.

In conclusion, the present study shows that not only lung metastasis but also lymph node metastasis occurs frequently in patients with myxofibrosarcoma. In addition, aggressive treatment for lymph node metastasis led to a satisfactory management of such patients. Further, detailed studies on the pathogenesis of remote metastasis of myxofibrosarcoma are highly desirable.
